# A high-throughput response to the SARS-CoV-2 pandemic

**DOI:** 10.1016/j.slasd.2024.100160

**Published:** 2024-05-16

**Authors:** Lynn Rasmussen, Shalisa Sanders, Melinda Sosa, Sara McKellip, N. Miranda Nebane, Yohanka Martinez-Gzegozewska, Andrew Reece, Pedro Ruiz, Anna Manuvakhova, Ling Zhai, Brooke Warren, Aliyah Curry, Qinghua Zeng, J. Robert Bostwick, Paige N. Vinson

**Affiliations:** Southern Research, Birmingham, AL, USA

**Keywords:** SARS-CoV-2, SARS, SARS2, SARS-CoV, MERS, COVID, COVID-19, Pandemic, Antiviral, High-throughput, HTS, BSL-3, Coronavirus, Drug discovery

## Abstract

Four years after the beginning of the COVID-19 pandemic, it is important to reflect on the events that have occurred during that time and the knowledge that has been gained. The response to the pandemic was rapid and highly resourced; it was also built upon a foundation of decades of federally funded basic and applied research. Laboratories in government, pharmaceutical, academic, and non-profit institutions all played roles in advancing pre-2020 discoveries to produce clinical treatments. This perspective provides a summary of how the development of high-throughput screening methods in a biosafety level 3 (BSL-3) environment at Southern Research Institute (SR) contributed to pandemic response efforts. The challenges encountered are described, including those of a technical nature as well as those of working under the pressures of an unpredictable virus and pandemic.

## 1. Introduction

### 1.1. Opening reflections

March 3, 2024 marked four years of working with SARS-CoV-2 at Southern Research Institute (SR) in Birmingham, AL. We now see our lives from the perspective of BC (before COVID) and AC (after COVID). While the economy and the general public have declared the pandemic over, it is worthwhile and even necessary to reflect on where we were in 2020. Were we prepared for such an event and how did we respond? We have this opportunity to learn what we did well and what we could have done better. The likelihood is high that those of us who experienced the COVID-19 pandemic will experience another pandemic in our lifetime. And impactful as COVID-19 was, the experience and outcomes could have been far worse. One only needs to look back to the historical 1918 influenza pandemic to understand what a high mortality rate, high transmission rate pandemic that caused 50 to 100 million deaths [1] looked like compared with 14 million deaths from COVID-19 [2]. Pondering the potential of another pandemic brings about a flood of questions that are valid considerations as we look ahead. What if the deadlier characteristics of the 1918 influenza were part of COVID-19? What if a highly pathogenic avian influenza retained its 50–95 % mortality rate, adapted to humans, and spread like seasonal flu? What if a coronavirus emerged that had the 30 % mortality rate of MERS-CoV (Middle East Respiratory Syndrome) [3] and the ability to spread through the human population like COVID-19? These scenarios are not beyond the scope of reason; they are possible and are the thoughts that keep infectious disease researchers awake at night. Other causes for concern include environmental factors unique to our modern era such as climate change, deforestation, and overdevelopment that are all accelerating human contact with insects, animals, and their pathogens [4]. Modern business practices and lifestyles make the emergence of novel pathogens in the near and distant future more likely.

With recent history as a guide, we reflect here on how Southern Research began preparing in the early 2000s for the demands that this pandemic brought to our laboratories. This perspective blends our views of the internal developments that were under our control and the external forces that were outside of our control as we were contributing to and witnessing this world-changing event. We describe how a small not-for-profit drug discovery organization in Birmingham, Alabama was ready on March 30, 2020, to support over 60 organizations in the race to develop a therapeutic for COVID-19. The background of that preparedness, the challenges encountered, and the ongoing considerations and developments will be discussed. Additionally, we describe the cell-based assays we have used to help drive SARS-CoV-2 drug discovery efforts and the timeline of these developments.

### 1.2. Before COVID

Southern Research brought a Biosafety Level Three (BSL-3) containment facility online in the mid-1980s to expand the types of infectious disease work that could be performed at SR. In the early 2000s, a group of virologists at SR set out to bring drug discovery processes like High-Throughput Screening (HTS) into the BSL-3 containment lab. The first step to prepare the lab space for that endeavor was upgrading liquid handling to a device that produced higher throughput than a 12-channel pipettor. A Biomek 2000 was moved into a biosafety cabinet inside the BSL-3 facility. Even though this initial step did not provide a full solution, it was the first of many that set SR on a path to develop the assays, the strategies, and the technologies that resulted in true high-throughput screening in BSL-3 containment [5].

### 1.3. NIAID support

Southern Research has a long history of federally funded infectious disease research. Targets have ranged from human immunodeficiency virus (HIV), orthopoxvirus, tuberculosis, and cytomegalovirus, among others. The combination of expertise, the formal establishment of an HTS Center, and access to a BSL-3 laboratory resulted in two pivotal funding awards from the National Institute of Allergy and Infectious Diseases (NIAID) that supported the development of high-throughput assays in the BSL-3 environment. Our first key success (in 2005) was a screen of a 100,000-compound library (Chembridge, Tripos) in Severe Acute Respiratory Syndrome Coronavirus (SARS-CoV) in 96-well plates (NIAID NO1-AI-30,047) [6]. The next step was to successfully miniaturize a BSL-3 assay to use 384-well plates. This was accomplished on NIAID N01-AI-15,449 (in 2007) where we screened 100,000 compounds (Chembridge) in Mycobacterium tuberculosis H37Rv [7]. This was almost immediately followed by a 384-well screen of 100,000 compounds (Chembridge) in SARS-CoV (2008) (NIAID NO1-AI-30,047, unpublished data).

In 2005 SR was selected as one of ten centers to serve in the Molecular Libraries Screening Center Network (MLSCN, U54 HG003917) as part of the Molecular Libraries Initiative of the NIH Roadmap for Medical Research [8]. Over time, many of the infectious disease screens were assigned to SR, particularly screens that required a BSL-3 facility. The MLSCN program supplied SR with stable funding and a steady supply of assays to develop and screen. This allowed SR scientists to further develop expertise in the area of infectious disease drug discovery. When the Roadmap progressed into the Molecular Libraries Probe Production Centers Network (MLPCN, U54 HG005034), SR was included as the Infectious Disease Specialty Center and served in this role for five years. During this time, SR received a wide range of infectious disease assays including viruses, bacteria, and fungi. Strategies were developed to run high-throughput assays with infectious agents in the BSL-2 as well as the BSL-3. A summary of these assays and screens are provided in Table 1 which includes the assay identification (AID) for the data uploaded to the PubChem Bioassay Repository (https://www.ncbi.nlm.nih.gov/pcassay).

Additional support followed the MLPCN grant in the form of an antiviral drug discovery U19 grant (U19AI109680). One of the first HTS campaigns under this grant was to screen for inhibitors of SARS-CoV with a 300,000 member in-house library composed of compounds purchased from commercial suppliers (Chembridge, Enamine, Life Chemicals, MicroSource, Enzo Selleck) and proprietary sets. In addition to coronaviruses, this grant included Alphaviruses, Flaviviruses, and Influenza. The HTS Center adapted or developed antiviral screening assays as well as several secondary assays to support chemistry efforts. Table 2 provides a cumulative list of antiviral and antibacterial screens inclusive of those performed under the MLSCN, MLPCN, U19, and in partnerships outside of these initiatives. In addition to the PubChem assay IDs listed in Table 1, many of the findings from the screens listed in Table 2 may be found in the references [6,7,9–23]. The efforts and accomplishments during this period built a solid foundation for 2020.

### 1.4. Streamlining the process

Two decades of support by NIAID provided both the funding and the infectious disease targets that allowed the HTS Center to develop screening strategies that improved efficiency. Many of the assay protocols transferred to the HTS Center contained multiple manipulations such as plating cells in advance and performing separate additions of test compound and virus. Some steps also called for a heterogenous assay design involving removal and replacement of well contents. Each manual manipulation contributes variability to the assay and reduces throughput by slowing the overall process and limiting batch size in contrast to a “mix-and-read” homogenous design. These comparisons of heterogenous and homogenous assays are not new and, indeed, are many times at the heart of assay transfer from a principle investigator’s laboratory to an HTS platform [24,25]. However, before altering the protocol, we would need to prove that the streamlining of the assay necessary to achieve the throughput goals would not negatively impact the results. The need to adapt assays to a design compatible with a high-throughput platform was not unique to the SR HTS Center but was nevertheless a hurdle to surmount, especially when serving the virology field that relies on assay designs that have been in place for multiple decades. Ultimately, we developed a robust general approach to performing cell-based antiviral assays in 384-well plates that remains in place today. This approach involves measuring cell viability using an ATP-dependent luciferase-based reaction (e.g. Promega CellTiter-Glo) resulting in a sensitive luminescence readout reflective of cytopathic effect (CPE) caused by the virus infection. The assay delivers consistent, robust results with Z’ values > 0.5 (Table 3). In addition to using automated devices such as bulk dispensers whenever possible and practical, other key details include 1) adding compounds to the plates as the first step which allows the use of high-throughput liquid transfer systems such as acoustic liquid handlers; 2) infecting the cells with virus in bulk, that is, adding the virus to the cells before adding to the plate which ensures a consistent well-to-well ratio of virus:cells; and 3) using a low multiplicity of infection (MOI) of virus in the assay which minimizes the need for pre-incubating cells with compound. More details for the coronavirus assays employing this approach are provided in the Methods section. To our knowledge, the SR HTS Center published the first high-throughput luminescence CPE assay to measure antiviral effect [23]. Small adaptations have been made, for example, preincubating neutralizing antibodies with the virus before adding the cells. And, for cells that do not exhibit CPE when infected by virus (but are still permissive and susceptible), the same set-up is used but a different measurement strategy is employed including using a reporter virus readout or measuring immunofluorescence with antibodies that recognize viral proteins [26].

**Fig. 1.**
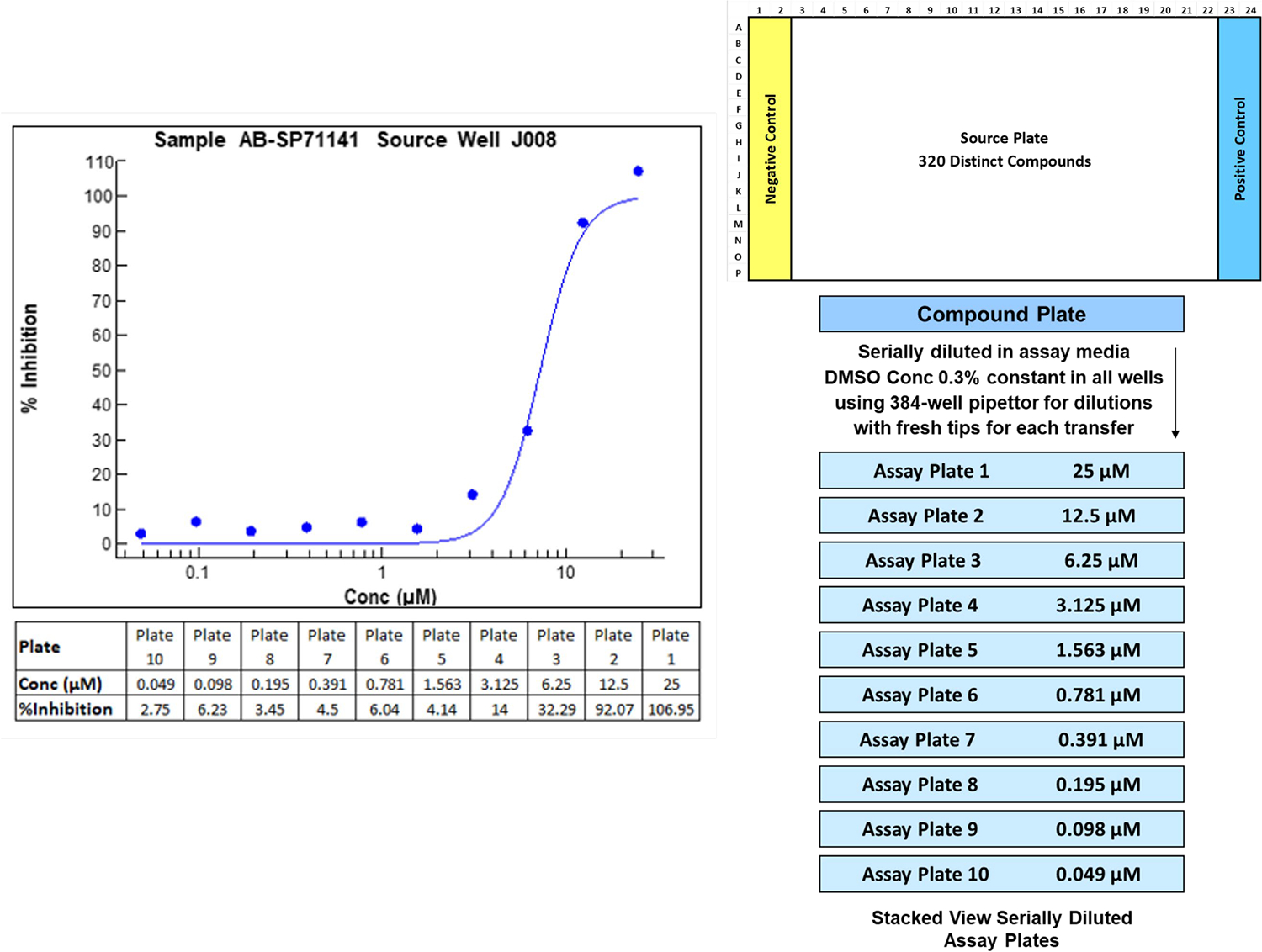
Illustration of the stacked plate concentration response experimental design. The format supports the creation of concentration response for 320 compounds when formatted in columns 3 – 22. Each plate contains a single concentration of each compound. This format provides a means to streamline performing a large number of concentration responses by formatting compounds into one plate and performing each dilution for all compounds simultaneously. Note: although the constraints of the fit model are 0 % - 100 % inhibition, experiments may produce readouts of 〈 0 % and 〉 100 % due to the ability of the biological signals to go outside the calculated average of the negative and positive controls.

Setting up the concentration response assay for compound confirmation experiments was initially performed by serially diluting selected compounds within a plate, which is a common practice among HTS laboratories. This resulted in testing 32 compounds per plate (10-point concentration-response) with each plate being set up separately on the liquid handler. We considered whether concentration response experiments could be made more efficient for large sets of compounds by setting up 320 compounds in one 384-well plate (columns 3 – 22) and stamping dilutions across a series of 384 well plates where each plate contains a specific dilution of the compounds (Fig. 1). We were confident of this design (referred to as a “stacked-plate” design) as we observed consistent control values across plates (average Z′ in the SARS-CoV assay was 0.65; in Influenza H3N2 it was 0.78), which was the primary requirement of combining data from different plates. It would also require a change in our data processing, a key component of our overall high-throughput capabilities. The changes in the liquid handling protocols to enable this new design were straight forward, however, changes in the data analysis strategy was not. SR’s HTS Informatics used ActivityBase (https://www.idbs.com) to manage the screening data. Although the ActivityBase platform could handle single dose data and in-plate dose response, it could not handle the stacked-plate dose response strategy we were developing. We had historically used the plate barcode to name the data file as it was exported from the plate reader as the key to connecting it to the plate record in the database. We had also created a method that prompted ActivityBase to import kinetic data where a distinct file was generated for each timepoint in the proper sequence. This was achieved by having the filename extension be a 3-digit numeric sequence rather than the typical txt or csv extension. This nomenclature was adopted to correlate the different plates used in the dilution sequence and link them to the same compound’s record in the stacked-plate dose response method. The change to the stacked-plate method addressed the bottleneck at this step and allowed the interrogation in dose response of many more of the single dose hits to ensure that we were not missing any promising compounds. Subsequently, ActivityBase released their XE module which enabled an automated correlation of distinct barcodes to their parent plate and resolved the need to modify output nomenclature to support this capability. The inclusion of this method further streamlined our approach and allowed the efficient testing of many compounds enabling the chemists to evaluate larger numbers of compounds for structure-activity relationship (SAR) determination. We continue to apply this approach for larger efforts (greater than approximately 100 compounds) when a concentration response design is required.

We and others have recognized the need for cytotoxicity counter-screen assays when evaluating compounds for antiviral activity. In antiviral assays, the cytotoxicity of a compound may be mistaken for an antiviral effect due to sub-lethal toxicity for the cells but sufficient toxicity to make the cells unable to support viral replication. Our process lends itself to efficiency of scale and, therefore, adding a cytotoxicity assay in parallel is an incremental increase in effort that can provide valuable decision-informing data in a timely manner. We employ this approach during confirmation of active compounds from HTS campaigns and when running concentration-response experiments to inform SAR chemistry efforts.

### 1.5. Early challenges and solutions

Not all assays behaved well. Respiratory syncytial virus (RSV) was one of those. The instability of RSV that is observed when generating stocks for experiments or vaccine preparations has been documented by others with notable features of RSV including inefficient replication and budding, unstable infectivity, and viral particles grown in vitro consisting mostly of large filaments [27–29]. We also experienced this challenge of instability while running an RSV antiviral screen. A virus stock was prepared, a titer was determined, and the assay validated. Once the screening commenced, the virus stock would degrade. This degradation resulted in insufficient infectious virus levels to run the assay. The initial response to this was to prepare a fresh stock, only to observe the same behavior. How the stock failed was interesting. The virus stock did not have a linear drop in titer over time. Rather, the titer would vary vial-by-vial such that one vial may have lost 0 %, 50 % or 100 % of the infectious virus when tested at the same time. After three rounds of virus stock failure, a different approach was necessary to achieve stability. We were aware of a strategy of using infected cells as a source of virus in the case of the retroviruses HIV and bovine immunodeficiency virus (BIV) [30]. Therefore, we applied that strategy to RSV and prepared frozen RSV-infected cells. A titer was determined for the frozen infected cells and the assay was validated using these cells [31]. The screening campaign was completed without any failures, including no observation of a reduction in virus titer [32,33]. The frozen infected cells were used throughout the project including hit identification, confirmation, and support for chemistry efforts. In 2023 these frozen infected cells from 2012 were used to prepare a new stock of virus. The cells from 2012 were still viable and virus was still infectious. This success was yet another lesson learned that would prepare us to be vigilant of the different aspects to consider when troubleshooting an assay, especially in the antiviral assay space.

### 1.6. Transferring to high containment

**Fig. 2.**
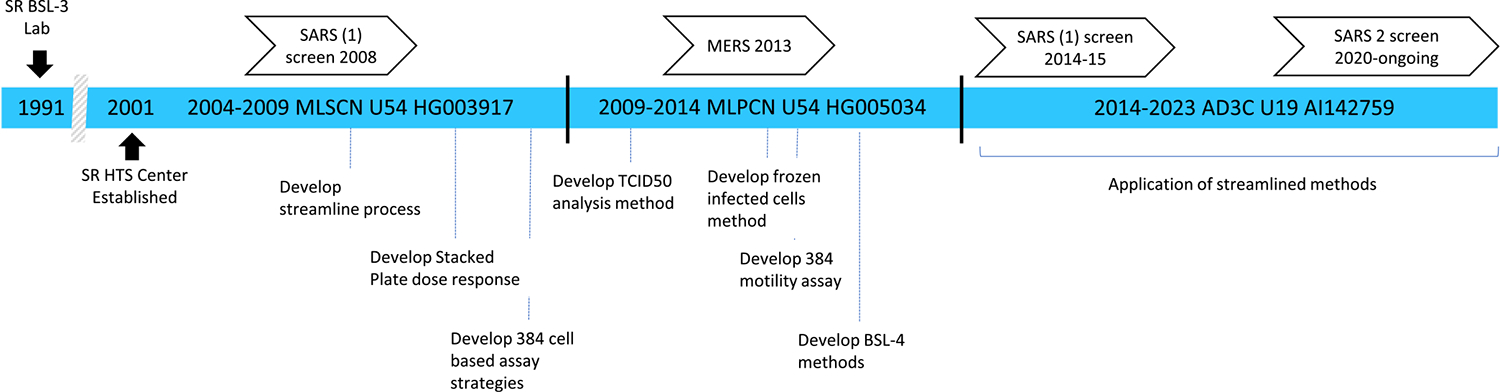
Learning timeline with key method developments and screens that helped prepare for the SR HTS response to the COVID-19 pandemic.

Most of the methods described above were first developed in the BSL-2 laboratory with materials compatible with that level of containment. After establishing miniaturized, streamlined assays under these conditions, the same strategies were applied to screens conducted in the BSL-3, resulting in true high throughput in a biocontainment laboratory. Although SR does not have a BSL-4 laboratory, we have collaborated with those that do to enable similar process improvements to researchers that screen within that higher level of containment. Partnering with University of Texas Medical Branch (UTMB), we guided their process to screen 10,000 compounds in both Ebola and Nipah antiviral screens [15]. These efforts established the feasibility of performing screening at all biosafety levels. Fig. 2 provides a timeline of grant funding, screening milestones, and streamlining developments.

### 1.7. A pandemic erupts

At the January 2020 SLAS meeting in San Diego, all eyes were on Wuhan, China. A novel coronavirus had emerged and was causing disease with a high mortality rate and the Chinese government had placed Wuhan under lockdown. Could this be contained like SARS-CoV, or would it spread? Were we watching the beginning of a global pandemic? No one knew. SR chose to hope for the best, but plan for the worst. Therefore, we started the process of obtaining the virus. We pursued acquisition of the virus from the World Reference Center for Emerging Viruses and Arboviruses (WRCEVA) which was in the process of growing a stock to distribute to researchers. This stock was prepared from the first diagnosed case of COVID-19 in the U.S. and would be known as the WA1/2020 strain and served as the reference virus for the pandemic. After many phone calls, e-mails, and much paperwork, we were ready to receive the virus stock. On March 3, 2020, a single vial of virus arrived from WRCEVA and we felt the sense of urgency to stay ahead of the viral spread in the population. With our background and experience with SARS-CoV, we found ourselves in a unique position; and due to the public funding that enabled this, we had an obligation to support efforts to develop therapeutics.

Like any coordinated effort, a prioritized list of necessary activities was generated:

1. Grow a working stock,
2. Determine the titer of the stock,
3. Develop a cytopathic effect (CPE) screening assay,
4. Validate the screening assay with compounds active against SARS-CoV,
5. Run a pilot screen using the SR bioactive library that includes FDA-approved drugs (approximately 4000 compounds) in single concentration, and
6. Follow-up the active compounds in concentration-response.

Even under the best of circumstances, each of these steps requires a minimum length of time to complete. The SR scientists working on this project felt extreme pressure to complete assay validation as quickly as possible to support efforts to develop a therapeutic. The HTS staff, including members in compound management, biologists, and our data management team, came together to move this project forward in spite of lockdowns, stay at home orders, school closings, and the massive public discord that began in March of 2020.

Indeed, the funding and efforts that had supported multiple projects over almost two decades were foundational to enabling the response of our group. In under 30 days, we had a characterized virus stock in the freezer and a validated screening assay running in the BSL-3. This was accomplished in March of 2020 when stay-at-home orders and lockdowns were sweeping across the United States and when global COVID-19 infections were rising exponentially as were COVID-19 deaths. And, although this was a coronavirus, it was novel, with no vaccines and no therapeutics. These were frightening times, but we moved ahead, and our screening assay was immediately put to use supporting drug discovery. As the toll of the pandemic continued to rise, an increasing number of clients and collaborators sent compounds to the SR HTS Center for screening. Even the nature of the sample types that we received reflected a sense of desperation. Disinfectant spray, unlabeled medicine bottles, and ointments were among the items that were sent. Several samples could not be handled due to their unknown nature. By September of 2020, there were 1 million confirmed deaths worldwide [34] and we did not have the tools to respond. In October of 2020, the delta variant emerged adding to the urgency [35].

As additional variants began emerging, new isolates were shipped to SR from the NIAID repository, BEI Resources. These included the Delta and Omicron variants for which additional CPE assays were developed. We also recognized the need for additional assay types. The SARS-CoV-2, as well as SARS-CoV and MERS-CoV CPE assays are performed in Vero cells, a kidney epithelial cell line derived from African green monkey. A positive characteristic of these cells is that they natively express angiotensin-converting enzyme 2 (ACE-2), the receptor for these coronaviruses. An undesirable feature is that they also express high levels of multi-drug resistance 1 (MDR1) protein (also referred to as P-glyco-protein 1, P-gp), an efflux pump that is responsible for removing xenobiotics from cells. The expression of MDR1 necessitates the addition of an inhibitor of that efflux pump into the assay if the compounds being tested are likely substrates, adding to the complexity of the assay. Addressing the first shortcoming, a human cell line would serve as a better model. However, we did not observe CPE in Calu-3, a lung adenocarcinoma cell line that expresses ACE-2, upon infection with the WA1/2020 isolate under conditions tested in our laboratory, a result reported by others [36] but not a universal observation. The laboratory of Ralph Baric at the University of North Carolina, Chapel Hill, developed a reverse-engineered system that generated a luciferase reporter version of WA1/2020 SARS-CoV-2 [37]. This reporter virus, along with A549 cells (human epithelial lung carcinoma) that recombinantly express ACE-2, provided another high-throughput assay that is complementary to the CPE assay. The cell line is human-derived, the assay measures virus levels, and there is negligible MDR1 expression in A549 cells negating the need for the addition of an efflux inhibitor. These assays are described in the Methods section.

### 1.8. Signs of hope

The first sign of hope came in December of 2020 with the release of the Moderna and Pfizer mRNA vaccines. But it took time to ramp up production, establish a large-scale vaccination campaign, and to start making an impact. By January 2021, 2 million people were dead; by April, 3 million; by July, 4 million; by November 5 million [34]. Work continued on vaccines as the emergence of variants caused concern that the existing vaccines would become less effective as the virus mutated. The urgency of developing a therapeutic continued and the screening workload experienced by the SR HTS staff remained high even as hope was beginning to emerge. Positive clinical trial results along with the November 2021 Owen et al. publication [38] that provided the structure of the antiviral component of Paxlovid (PF-07321332 or Nirmatrelvir) resulted in requests by clients to add it as a reference compound when screening.

**Fig. 3.**
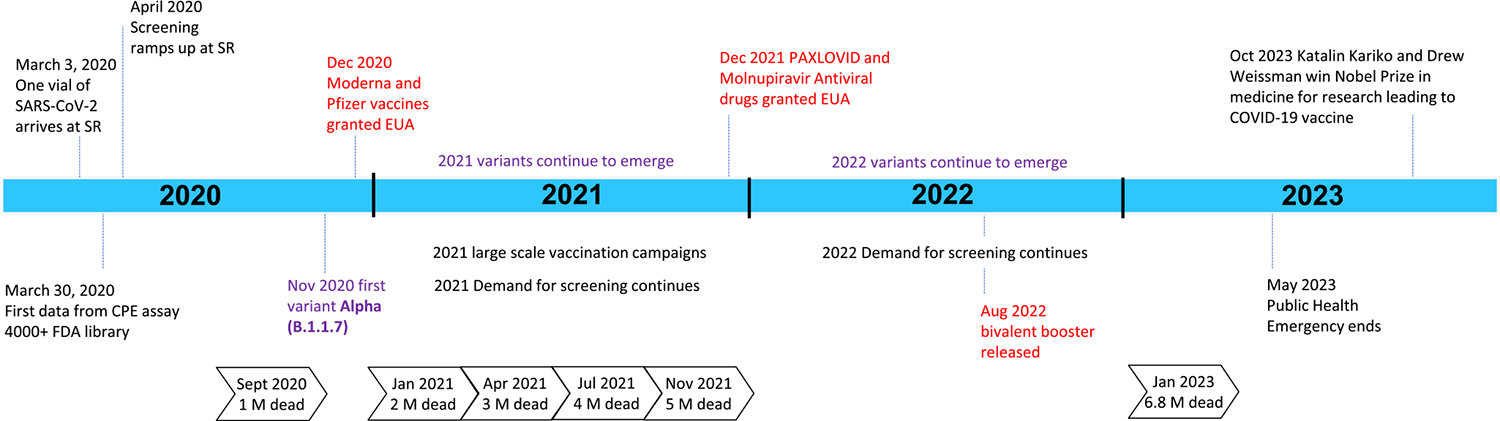
Timeline showing milestones in the SR HTS’ SARS-CoV-2 screening efforts, therapeutic developments, and death tolls. Significant therapeutic developments are shown in red text. The introduction of variants into the population are shown in purple text.

In December 2021 two antiviral drugs were released for emergency use, Paxlovid and Molnupiravir [38,39]. These gave us additional clinical tools for the battle with COVID-19. The rate of infection and death from COVID-19 started to slow as herd immunity from vaccination or infection/recovery became widespread. With the addition of therapeutics, the death rate was further reduced. But COVID-19 was not done with us yet; variants continued to emerge and spark new waves of infection even among fully vaccinated people. We also learned that COVID-19 did not produce long-term immunity which was also contributing to ongoing waves of infection. In August 2022, a bivalent booster vaccine was released to address the variant issues. January 2023 the milestone of 6.8 million confirmed deaths was reached [34]. Recently the World Health Organization (WHO) recalculated the number of COVID-19 deaths using excess deaths calculations placing the number of COVID-19 deaths at 14 million globally [2]. The discussed events of the COVID-19 pandemic are shown in Fig. 3.

## 2. Results and discussion

**Fig. 4.**
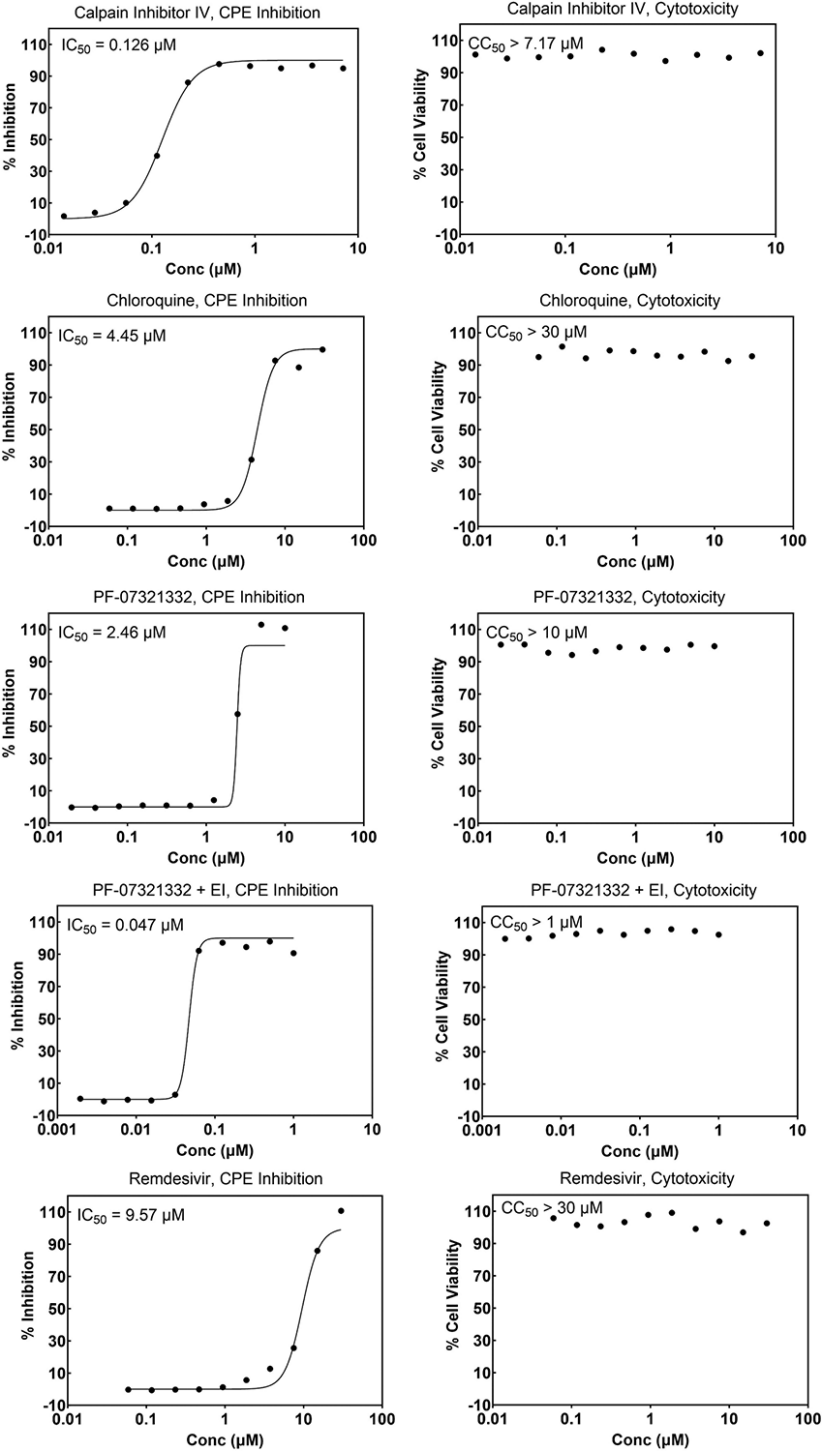
Concentration response graphs for reference compounds run in the cytopathic effect assay (CPE) and corresponding cytotoxicity. Vero E6 cells with enhanced ACE-2 expression were infected with the WA1/2020 isolate of SARS-CoV-2. Inhibition of CPE is expressed as the 50 % inhibitory concentration (IC_50_) and cytotoxicity is expressed as the 50 % cytotoxicity concentration (CC_50_). EI = efflux inhibitor (CP-100356); Conc = Concentration.

**Fig. 5.**
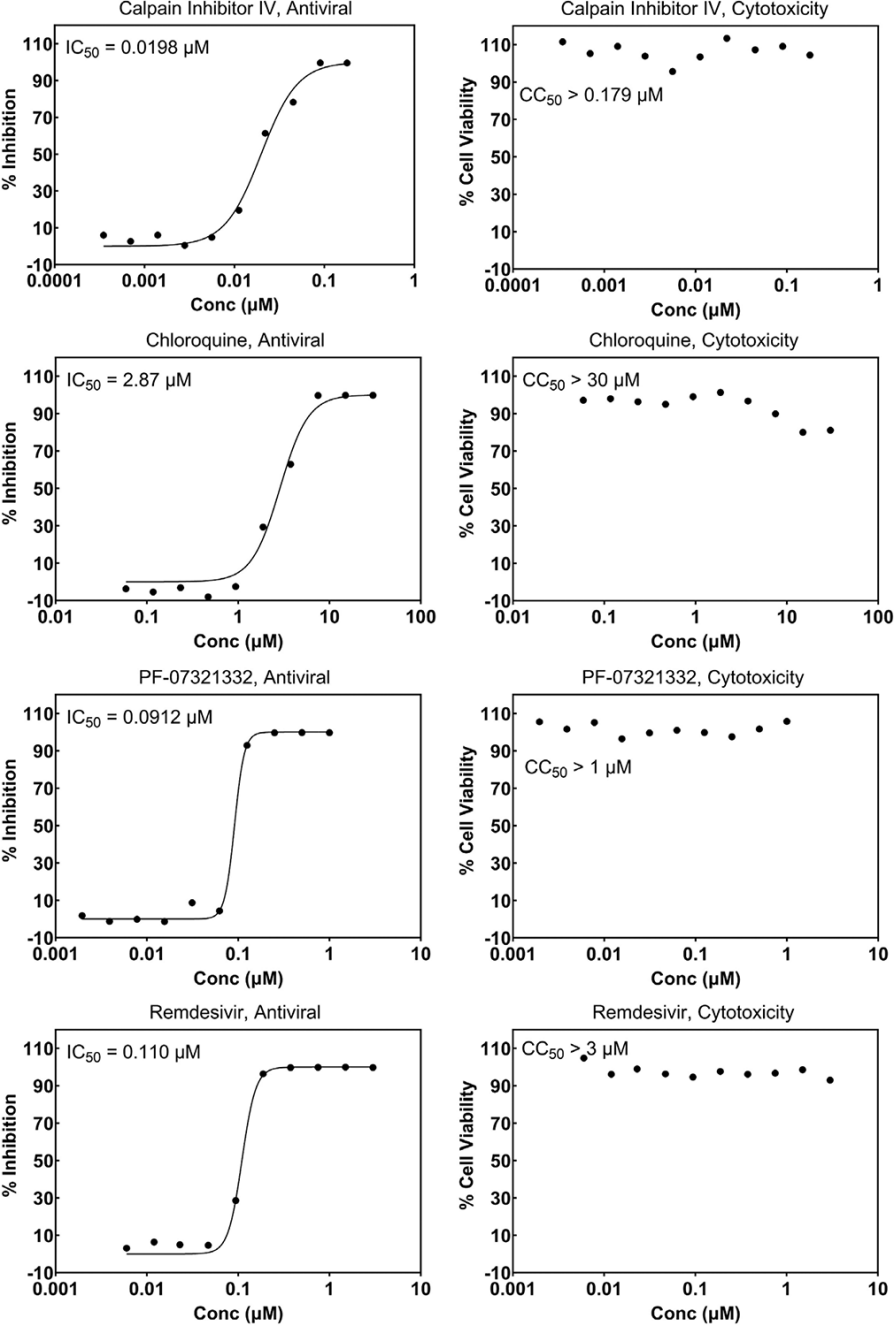
Concentration response graphs for reference compounds run in the SARS-CoV-2 NanoLuc reporter virus assay in A549 cells that recombinantly express ACE-2. The left panel shows the antiviral assay results and the right panel the corresponding cytotoxicity assay results. Inhibition of reporter virus signal is expressed as the 50 % inhibitory concentration (IC_50_) and cytotoxicity is expressed as the 50 % cytotoxicity concentration (CC_50_). Note the exclusion of efflux inhibitor due to the lack of significant levels of MDR1 in A549 cells. Conc = Concentration.

**Fig. 6.**
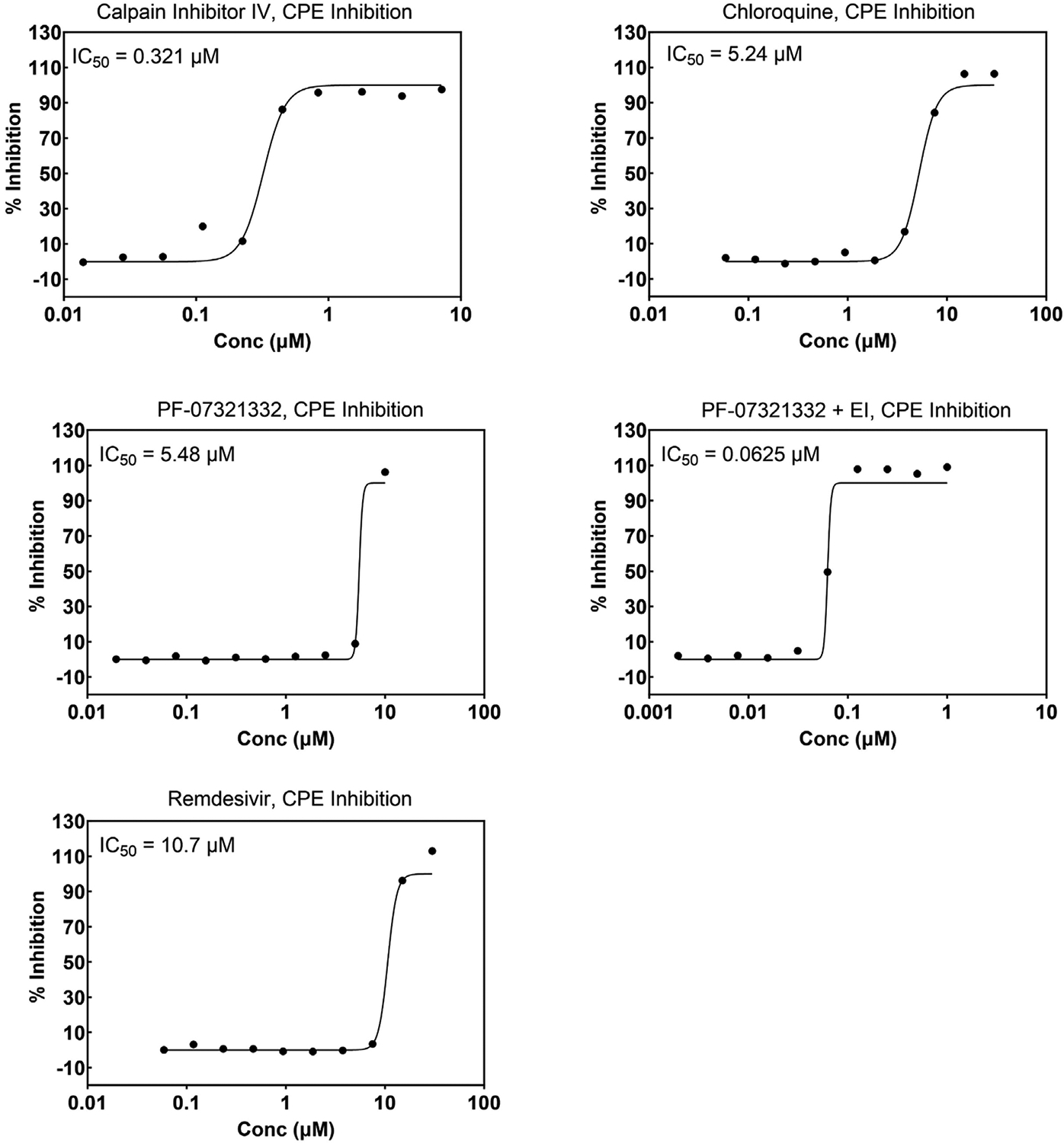
Concentration response graphs for reference compounds run in the cytopathic effect (CPE) assay. Vero E6 cells with enhanced ACE-2 expression were infected with the Toronto-2 strain of SARS-CoV. Inhibition of CPE is expressed as the 50 % inhibitory concentration (IC_50_). As illustrated in Fig. 4 (same cell line), none of the compounds tested exhibited measurable cytotoxicity. EI = efflux inhibitor (CP-100356); Conc = Concentration.

**Fig. 7.**
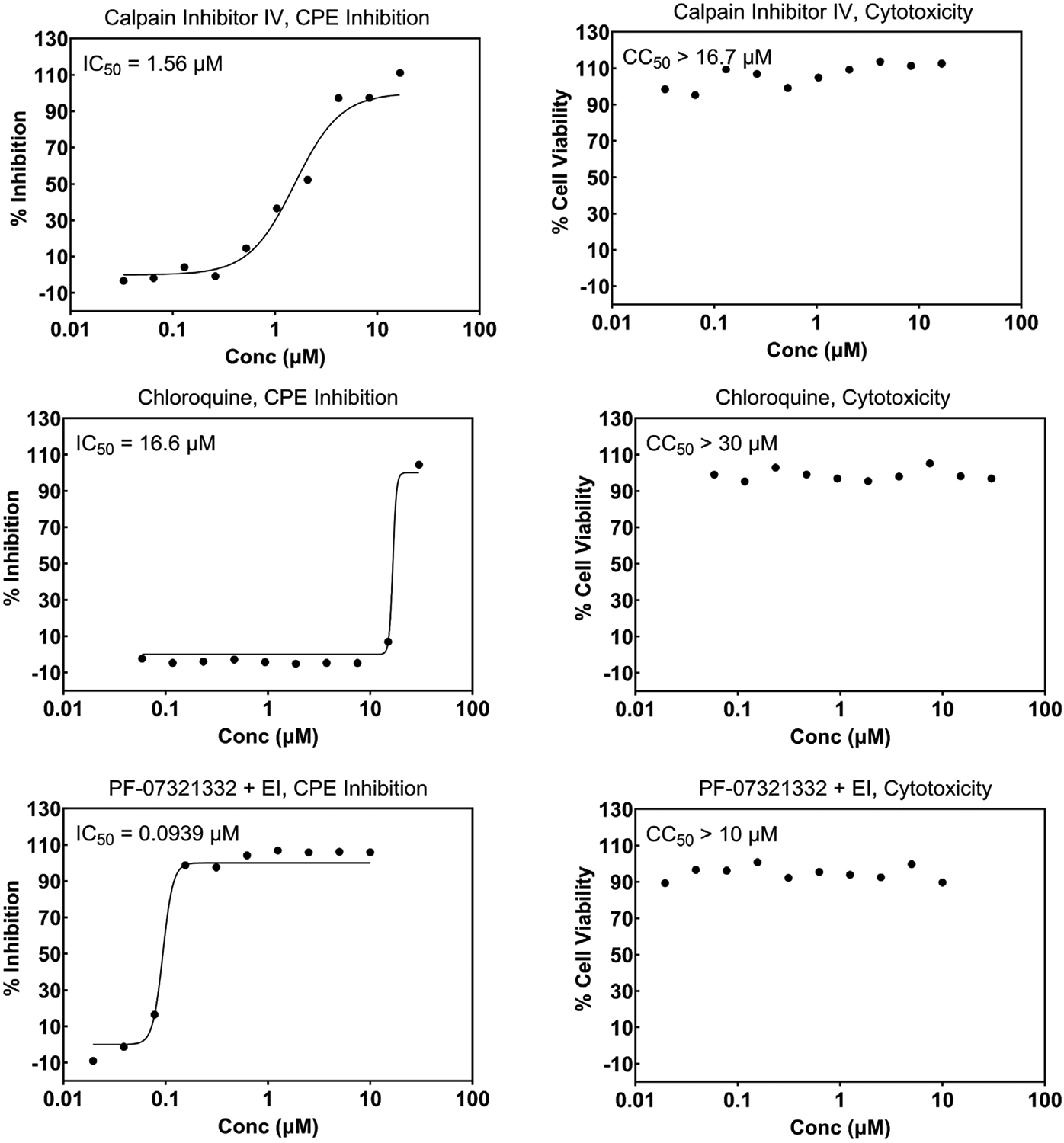
Concentration response graphs for reference compounds run in the cytopathic effect (CPE) assay in Vero CCL-81 cells infected with MERS-CoV and corresponding cytotoxicity. Inhibition of CPE is expressed as the 50 % inhibitory concentration (IC_50_) and cytotoxicity is expressed as the 50 % cytotoxicity concentration (CC_50_). EI = efflux inhibitor (CP-100356); Conc = Concentration.

The first SARS-CoV-2 virus sample was received at SR on March 3, 2020, and an internal effort was initiated immediately to develop screening assays as described in the introduction. Internally developed compounds known to inhibit SARS-CoV were tested early during assay implementation and, in most cases, crossover activity was observed. As more compounds were discovered and made public to be active against SARS-CoV-2, these were added to help monitor the assay performance. Figs. 4 and 5 show representative graphs of Calpain Inhibitor IV, Chloroquine, PF-07321332, and Remdesivir in the Vero CPE assay and A549 reporter assay, respectively, along with corresponding cytotoxicity data. The differing potencies can be attributed to the different cell types as well as the readout (CPE vs reporter virus levels). The effect of the addition of efflux inhibitor is demonstrated for PF-07321332 in the CPE assay. Fig. 6 shows data for the same compounds in the SARS-CoV CPE assay in Vero cells which demonstrates comparable activity of these antivirals for the two coronaviruses. Similarly, Fig. 7 shows data for Calpain Inhibitor IV, Chloroquine, and PF-07321332 (+ efflux inhibitor) for MERS-CoV. The data sets shown were selected to represent the average potency value that has been observed over numerous runs. A summary of the performance of these assays and average potency values observed, including for CPE assays for the Delta and Omicron variants of SARS-CoV-2, are shown in Table 3. The values fall within the range of published values [38,40–43] and the quality is excellent. Most inhibitors exhibit highly reproducible values. In the MERS-CoV assay, Chloroquine does not always reach full inhibition at the top concentration tested (30 μM) and, therefore, the average potency value shown is considered approximate. There is also a notable lower potency (higher IC_50_) for Calpain Inhibitor IV in the MERS-CoV assay compared to all of the others performed. Comparisons of this inhibitor’s activity across different coronaviruses could not be found in the literature and we have not further investigated this difference to be able to attribute it to a specific feature of the virus or assay. The MERS-CoV CPE assay is the only one that is performed over four days, rather than three, which may contribute to this difference. The higher variability of the IC_50_ for PF-07321332 + efflux inhibitor is discussed below. Note that we use the conventions of half-maximal inhibitory concentration (IC_50_) as the antiviral potency of a compound and 50 % cytotoxic concentration (CC_50_) when describing the cytotoxic effect of a compound.

**Fig. 8.**
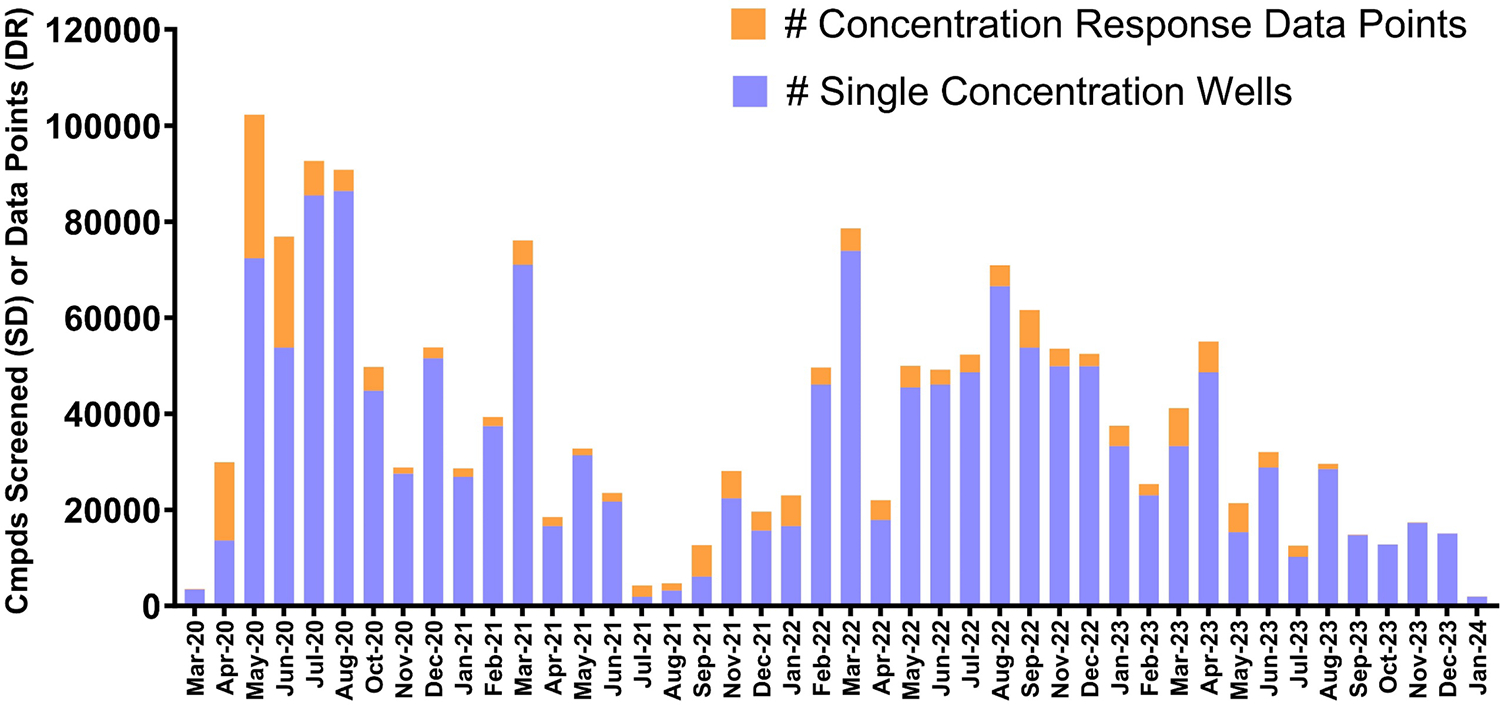
Graph represents the number of compounds screened in single concentration (which includes assay ready plates) and the number of wells screened in concentration response in SARS-CoV-2 cell-based assays at Southern Research between May 2020 and January 2024. This represents a total of approximately 1.5 million antiviral data points. Note that the numbers do not include the parallel cytotoxicity assay data.

On March 30, 2020, the validated SARS-CoV-2 assay was used to interrogate 4000+ FDA-approved and other known biologically active compounds most of which were previously purchased from Selleck, Enzo and Microsource. Over the next 3+ years, this assay would generate millions of data points and support drug discovery efforts for over 60 clients and collaborators. A total of over 10,283 compounds have been run in a dose-response format and over 1429,000 single-dose conditions have been run, not including the parallel cytotoxicity that is performed as a dose response counter-screen (Fig. 8). It is tempting to correlate the peaks and valleys of our screening volume to the surges and quells of infection rates, however, it is more accurately described as a conglomerate of demand and logistical factors including the requirement to shut down a BSL-3 laboratory at least once a year for maintenance. The majority of the screening volume originated from commercial clients and, therefore, much of the contribution from our group’s screening is not publicly acknowledged. However, in our view, this does not negate its significance. Two examples of our collaborative efforts with the National Center for Advancing Translational Sciences (NCATS) may be found in peer-reviewed publications [44,45].

Like the expanded scientific community, we learned lessons along the way and constantly questioned what our next development should be. As mentioned in the introduction, we were cognizant that Vero cells are not an ideal system. They are not human in origin (monkey kidney cell line) and have low expression of proteases such as TMPRSS2 and trypsin, affecting the mechanism of entry of the virus into the cells [46]. In addition, the variants that were emerging exhibited mutations that influenced the interaction between the virus receptor binding domain and ACE-2 [47]; this led to the question of whether or not the variant we used in an assay would be representative of the future circulating variant (s) that tested compounds would need to be active against. In both of these cases, we chose to not let “perfection be the enemy of excellence”. In other words, we ensured the assays we provided to collaborators and clients performed in a robust and reproducible manner and considered our concerns with this in mind. Introducing the SARS-CoV-2 NanoLuc reporter virus enabled a move to a human cell line, with its own limitations (recombinantly expressed ACE-2 and an engineered form of the virus). We also relied on the collaborators and clients that we serve to steer our developments as they deemed necessary for the antiviral mechanisms they were addressing. This approach led to our addition of Delta and Omicron variants, although the WA1/2020 isolate continues to be the assay primarily performed. These types of challenges are not unusual for the selection of a primary assay that will inform the decision of whether or not to progress compounds to more complex assays. As in any drug discovery program, one wants the primary assay to serve as a reliable means of prioritizing compounds and we believe this suite of assays achieved that aim. In addition, the availability of SARS-CoV and MERS-CoV assays provides complementary data to determine whether or not tested compounds may exhibit pan-coronavirus protection.

**Fig. 9.**
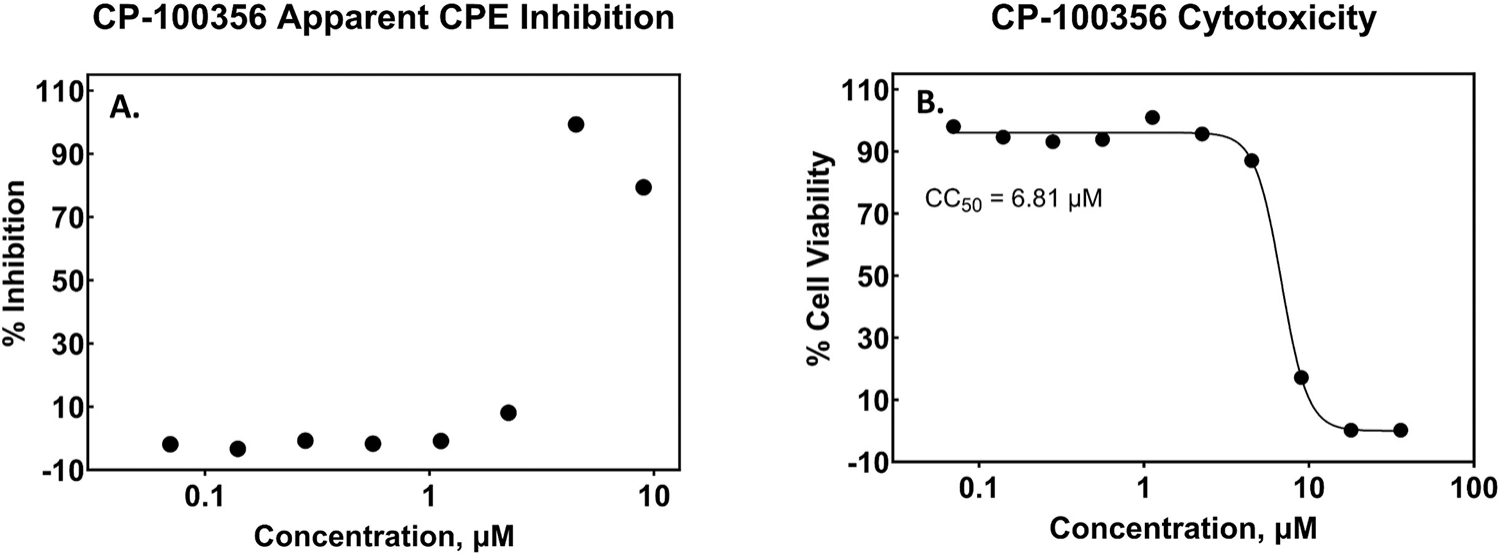
Concentration response of the MDR1 inhibitor CP-100356. A.) The measured effect in the SARS-CoV-2 CPE assay performed in Vero E6 cells. B.) The cytotoxic effect of CP-100356 observed in Vero E6 cells.

Another notable feature of the Vero assays that we observed was the effect of the MDR1 inhibitor CP-100356. Because Vero cells express MDR1, there were occasional requests to include this additional component into the assay along with the compounds to block their efflux out of the cell through this channel. The IC_50_ of CP-100356 inhibition of MDR1-mediated transport of Calcein-AM has been determined to be 0.5 μM [48]. Therefore, final concentrations of 1 – 2 μM CP-100356 were added to assays when this mechanism of compound expulsion from the cells was suspected. We noticed a decline in assay performance when this efflux inhibitor was added and, in some cases, an effect on cell viability in the parallel cytotoxicity assay. This was not a consistent occurrence. In the cases where cytotoxicity was not observed, a shift to higher potency (lower IC_50_) occurred, as expected (Figs. 4, 6, 9 and Table 3). And, the potency of PF-07321332 + CP-100356 (1 μM) agreed well with a published report of the potency of the same compound in Vero cells with MDR1 genetically knocked out [49], confirming the desired effect of the efflux inhibitor. To better understand the effect of CP-100356, we performed a concentration response of only this compound in both the SARS-CoV-2 CPE and the Vero E6 cytotoxicity assays. The results are shown in Fig. 9. The CC_50_ of CP-100356 was measured to be 6.81 μM under the conditions of our assay. At 1.0 μM there is no observed effect on either CPE or cytotoxicity indicating that this is an acceptable concentration at which to run the assay. However, as can be observed in the graphs, 1.0 μM is on the cusp of where an effect is starting to occur and small changes in volume or other factors such as batch-to-batch variability in CP-100356 could tip the scale, resulting in a misleading interpretation of an antiviral effect. The apparent inhibition of CPE at 4.5 μM (99 %) is likely associated with the cytotoxic effect of CP-100356, presenting an example of the importance of performing parallel cytotoxicity assays for any compound (regardless of the presence or absence of efflux inhibitor) as well as reinforcing the need to understand the effect of any additional components to an assay.

### 2.1. Closing reflections

As of April 1, 2024, there have been 775.26 million confirmed cases of COVID-19 and 7.04 million confirmed deaths globally due to this infectious disease [34]. Recent data from WHO put the death toll at over 14 million, using “excess deaths” analysis. While the response to this public health emergency was rapid and heavily resourced at levels not possible in the past, the death toll was still alarming. The response included ways and means to effectively screen potential new therapeutics, both small molecules and biologics. In the case of target-based therapeutic development, BSL-1 and BSL-2 laboratories pivoted to run biochemical assays for viral targets such as RNA-dependent RNA polymerase (RdRp) and SARS-CoV-2 main protease (Mpro or 3CL protease). BSL-2 laboratories also scaled up to increase capacity of virus testing in the community. As with any drug discovery program, the need for high-throughput assays in a more translatable context are necessary for promising compounds to progress for further profiling and development. We already had experience with SARS-CoV in developing assays for screening in a BSL-3 biocontainment laboratory. Due to the development of high-throughput assays for other coronaviruses requiring BSL-3 containment, the SR HTS Center was well-positioned for a rapid and robust response to this new threat. When first embarking on an assay development effort for SARS-CoV-2, we were fortunate that our starting point, the protocol previously developed for SARS-CoV, required very few modifications. Thus, the SARS-CoV-2 assay came online quickly and was used to confirm hits from biochemical assays and set up screens to further advance active compounds. This was significant because the assay was cell-based and used the pandemic isolate in an antiviral assay, not a less pathogenic surrogate. It gave us confidence to move forward with large scale screening.

Our work represents a piece of the complex puzzle that was worked on by multiple groups to bring effective therapies to the public. Despite almost two decades of experience, HTS in a biocontainment lab (BSL-3) still brings numerous challenges. The first is that much of the automation equipment in common use for screening cannot be used in the BSL-3. The space constraints of the lab limit the size of equipment; any equipment that creates an aerosol needs to be located in a biosafety cabinet or Bio-Bubble. Service support is also a limitation as most equipment needs to be decontaminated and taken out of the BSL-3 for service or maintenance. And finally, a biocontainment lab is a demanding environment for the humans who work in that space due to the entry/exit protocols, enhanced personal protective equipment (PPE) that must be worn, and the stress of working with a pathogen that is not well characterized and is responsible for millions of deaths.

If we expand our view and look at the impact of SARS-CoV-2 around the world, the same trends were evident in most developed countries. At the time, infectious disease researchers, health care professionals, and epidemiologists were learning and adjusting in real time. Until effective vaccines were available starting in December of 2021, the virus spread rapidly and had a high mortality rate among the elderly and people with underlying health conditions; healthcare disparities were magnified. What initially appeared to be less severe effects of COVID-19 in developing nations, such as in Sub-Saharan Africa, eventually became better understood as potential under-reporting of infections [50,51]. Although the population in many of these regions is younger and more robust, reduced access to healthcare offset this advantage, resulting in a fatality rate similar to that of higher income nations [51]. At the end of 2021, harsh COVID-19 restrictions were still in place in China and no actions were taken to prepare the population for the rollback of these restrictions. After significant political unrest related to the COVID-19 restrictions, the Chinese government abruptly rolled back the restrictions on December 7, 2022. This set off another surge of infections and death [34,52]. At the end of December 2023, the weekly confirmed cases in China peaked at 40 million [34]. This impacted the Chinese economy and sent shockwaves through the global supply chain again.

Even though the United States federal Public Health Emergency for COVID-19 expired in May 2023, the development of novel therapeutics continues to address emerging variants of concern and potential resistance to existing vaccines and small molecule therapies. The COVID-19 experience has also brought the reality of future pandemics to the forefront, emphasizing the need for a rapid response with urgency and focus. With that in mind, strategies are being put in place to develop updated booster shots for annual immunizations, similar to how influenza is handled. Work also continues to develop additional therapeutics to address the possibility of arising drug resistant variants. In July of 2021, NIAID posted a U19 funding opportunity entitled “Emergency Awards: Antiviral Drug Discovery (AViDD) Centers for Pathogens of Pandemic Concern” that solicited proposals for the discovery and development of antivirals targeting SARS-CoV-2 and other RNA viruses with pandemic potential. In May of 2022, NIAID announced the award of this grant totaling approximately $577 million to establish nine antiviral drug discovery centers for pathogens of pandemic concern [53], demonstrating a continued commitment to funding efforts toward pandemic preparedness in an ever-changing landscape. Coordinated efforts such as that described in the European Centre for Disease Prevention and Control (ECDC) technical report entitled “Public health and social measures for health emergencies and pandemics in the EU/EEA: recommendations for strengthening preparedness planning” demonstrates the awareness of the need to implement non-pharmaceutical measures in social and public health as part of pandemic preparedness planning [54].

During the last four years, we were impressed by the range of organizations (from individuals and small startups to academic institutions and large pharmaceutical companies) involved in the search for a therapeutic as well as the sample types that were sent to us, several of which could not be tested due to a lack of identifiable information. This is a testament to the feelings of desperation during the first few months of the pandemic and the attitude of “try anything” which included deploying mRNA vaccines. Research and development of mRNA as a therapeutic had been in the works since the late 1990s but had not been approved by the FDA. The horror of disease and death brought on by the pandemic was the event that gave mRNA vaccines a chance to prove their value. The good that came out of the pandemic is that we have built the infrastructure for rapid vaccine and therapeutics development. This foundation can continue to be built upon by the next generation of scientists and public health funding to ensure preparedness for future infectious disease threats across the pathogen spectrum.

## 3. Materials and methods

### 3.1. Virus strains and cell lines

The viruses and cell lines utilized in the assays are summarized in Table 4.

### 3.2. Cell culture

For Vero E6 and Vero CCL-81 cell lines, the cell culture medium consisted of DMEM (Corning 10–013-CM) supplemented with 10 % heat-inactivated FBS (PEAK PS-FB4) and cells were sub-cultured twice a week at a split ratio of 1:2 to 1:5 using standard cell culture techniques. A549 cells expressing ACE-2 were grown in DMEM supplemented with 20 % heat-inactivated FBS, 1 % non-essential amino acids, 100 μg/ml blasticidin and split 1:6 every three days with removal of blasticidin from the medium one passage before using the cells in the assay. On the day of the assay, cells were harvested and resuspended in assay medium consisting of DMEM, 2 % heat inactivated FBS, 1 % Pen Strep (Corning 30–002-CL), 1 % HEPES buffer (Corning 25–060-CL). Total cell number and percent viability determinations were performed using a Luna cell viability analyzer and trypan blue dye exclusion. Cell viability was required to be greater than 95 % for the cells to be utilized in the assays.

The day of each assay, a pre-titered aliquot of virus was removed from the freezer (−80 °C) and allowed to thaw to room temperature in a biological safety cabinet. The virus was diluted in assay medium to achieve a 100x concentration. This was added to cells to achieve a 1x concentration of virus (approximate MOI of 0.002).

### 3.3. Compound preparation

Reference compounds and efflux inhibitor were purchased from commercial sources. These included the dual MDR1 (P-gp)/BCRP inhibitor, CP-100356 (MedChemExpress, Cat. No. HY-108,347), Remdesivir (Selleck Chemicals, Cat. No. S8932), Chloroquine phosphate (Selleck Chemicals, Cat. No. S4157), Calpain Inhibitor IV (EMD Milli-pore, 208,724), and PF-07321332 (MedChemExpress, HY-138,687). For compounds dissolved in DMSO, the compound stock solution was serially diluted resulting in 10 concentrations, each at 333X of the final assay concentration. The dilution strategy was dependent on estimated potency of the compound(s) or as specified by the collaborator. Using a Beckman Echo 550 or 555, 90 nL of the DMSO solutions were transferred from the source plate to the assay plate(s), including matching amounts of DMSO in control wells not containing compound. Plates were sealed and frozen at −80 °C and at this point these are considered Assay Ready Plates (ARPs) irrespective of whether they were prepared at SR or prepared by the client and shipped to SR. For assay set up the plates were brought to room temperature, seals removed and, 5μL of assay media added to each well. All wells were solvent-matched.

Test agents dissolved in buffer, such as antibodies, were diluted in assay medium and serially diluted resulting in 10 concentrations at 6X the final assay concentrations. Using a Beckman FX liquid handler, 5 μL were transferred to the antiviral and cytotoxicity test plates. All wells were solvent-matched.

### 3.4. Cytopathic effect (CPE) assay

The assay plates containing compound dilutions were passed into the BSL-3 facility along with two flasks of cells at 160,000 cells per mL. One flask of cells was used to plate the cell controls (100 % inhibition of CPE) and contains no virus. The other flask was batch inoculated with the variant being tested at a multiplicity of infection (MOI) of approximately 0.002 and stirred at 200 RPM for approximately 10 min. This had previously been determined to result in 5 % cell viability 72 h post-infection with SARS-CoV in Vero E6 cells and 96 h post-infection with MERS-CoV in Vero CCL-81. A 25 μL aliquot of cells only were plated first in columns 1 and 2 followed by the addition of virus-inoculated cells in columns 3–24 of the assay plates. The wells in columns 23–24 contained only virus-infected cells for the 0 % inhibition of CPE controls. The resulting volume was 30 μL in each well. After incubating plates at 37 °C/5 % CO_2_ and 90 % humidity for 72 h (SARS-CoV-2 and SARS-CoV) or 96 h (MERSCoV), 30μL of CellTiter-Glo (Promega) was added to each well without removing media. Luminescence was read using a BMG CLARIOstar plate reader following incubation at room temperature for 10 min to measure cell viability. Plates were sealed with a clear cover and surface decontaminated prior to luminescence reading.

### 3.5. Nanoluc reporter virus (NLRV) assay

On the day of assay, A549-ACE-2 cells were harvested in DMEM supplemented with 2 % heat inactivated FBS, 1 % HEPES, 1 % Pen/Strep. Assay ready plates pre-drugged with test compounds were prepared in the BSL-2 lab by adding 5μL assay media to each well. The plates and cells were then passed into the BSL-3 facility. As with the CPE assay, 25 μL of cells only were dispensed to columns 1–2 (no test compounds) of each plate for the cell only 100 % inhibition controls. Working stock of SARS-CoV-2 nanoluciferase reporter virus (NLRV) passaged five times in A549 cells expressing ACE-2 was diluted to 100X in media then diluted 1 to 100 into a cell suspension containing 160,000 cells per mL (MOI approximately 0.002) and stirred at 200 RPM for approximately 10 min. A 25 μL aliquot of virus-inoculated cells was added to each well in columns 3–24 of the assay plates. The wells in columns 23–24 do not contain test compounds, only virus-infected cells for the 0 % inhibition controls. This resulted in a total volume of 30 μL/well. After incubating plates at 37 °C/5 %CO2 and 90 % humidity for 72 h, 30 μL of NanoGlo (Promega) was added to each well. Note that the amount of NanoGlo substrate added to the buffer is reduced 10-fold to reduce the intensity of the signal which can saturate the detector. Luminescence was read using a BMG CLARIOstar plate reader (bottom read) following incubation at room temperature for 20 min to measure luciferase activity as an index of virus titer. The time between substrate addition and read time must be tightly controlled as the signal does not stabilize like the Glo-based reagents. The signal increases over 10–15 min then starts to decrease. Plates were sealed with a clear seal and surface decontaminated prior to luminescence reading.

### 3.6. Cytotoxicity assay

Compound cytotoxicity was assessed in a BSL-2 counter screen as follows: Host cells in media were added in 25 μL aliquots (4000 cells/well) to each well of assay ready plates prepared with test compounds as above. Cells only (100 % viability) and cells treated with hyamine at 100 μM final concentration (0 % viability) served as the high and low signal controls, respectively, for cytotoxic effect in the assay. DMSO was maintained at a constant concentration for all wells as dictated by the dilution factor of stock test compound concentrations. After incubating plates at 37 °C/5 % CO_2_ and 90 % humidity for 72 h, 30 μL CellTiter-Glo (Promega) was added to each well and plates incubated for 10 min at room temperature. Luminescence was then read using a BMG PHER-Astar plate reader to measure cell viability.

### 3.7. Data analysis

For all assays the raw data from plate readers were imported into ActivityBase v.9.7 (IDBS) where values were associated with compound IDs and test concentrations.

For the antiviral CPE reduction and the NanoLuc reporter assays, raw signal values were converted to% CPE reduction by the following formula:

$$\%\text{antiviral}\;\text{effect}=100\times \frac{\left(\text{test}\;\text{compound}\;\text{value}-{\text{mean}}_{\text{infected}\;\text{cell}\;\text{controls}}\right)}{\left({\text{mean}}_{\text{uninfected}\;\text{cell}\;\text{controls}}-{\text{mean}}_{\text{infected}\;\text{cell}\;\text{controls}}\right)}$$

For the cell viability assay measuring compound cytotoxicity,% cell viability was calculated as follows:

$$\%\text{viability}=100\times \frac{\left(\text{test}\;\text{compound}\;\text{value}-{\text{mean}}_{\text{low}\;\text{signal}\;\text{control}}\right)}{\left({\text{mean}}_{\text{high}\;\text{signal}\;\text{control}}-{\text{mean}}_{\text{low}\;\text{signal}\;\text{control}}\right)}$$

IC_50_ (inhibitory concentration) and CC_50_ values were calculated from a four-parameter logistic fit of data using the XLFit module of ActivityBase.

## Figures and Tables

**Table 1:** Antiviral and antibacterial assays performed and their corresponding PubChem Summary AID (assay ID). Data may be searched at https://www.ncbi.nlm.nih.gov/pcassay/. Collections obtained from the Molecular Libraries Small Molecule Repository were the source of compounds for screens listed under the MLSCN and MLPCN headings.

SR Assay	Cumulative Rows uploaded	PubChem Summary AID
TB (Kinase Library)	23,821	2842
TB (CB2)	100,685	1949
TB (Prestwick)	1,117	1332
MLSCN Assay	Cumulative Rows uploaded	PubChem Summary AID
Influenza H5N1	95,520	740, 806
Arbovirus	196,156	1251, 1250
E. coli	186,112	552, 710, 573, 617, 638, 635
mtb H37Rv	215,397	1626
MLPCN Assay	Cumulative Rows uploaded	PubChem Summary AID
WNV	290,458	1635
RSV	316,542	2440
VEE	349,796	588723
NS1A H1N1	340,920	504399
HIV Viral infectivity cytoprotection*	702	1991*
HSV	215,049	504781*
TB Intein*	137	2223*
TB Inhibition 7h9	333,111	449775
TB SL beta lactam	330,831	434999
TB Non Replicating	329,133	488929
E.Coli BCBI	337,322	488970
PhoP (Salmonella Typhi)	313,697	1864
PhoP (Salmonella Typhimurium)	307,971	1874
SV40	30	1909
Dengue	10,240	540333

* Summary AID belongs to other center, SR performed secondary assays

**Table 2:** Cumulative number of compounds screened in cell-based assays, spanning various grants and contracts and including various iterations and conditions. The number of compounds screened varies depending on scope, budget, and desired library content of the project. Numbers are inclusive of screens listed in Table 1.

BioSafety Level	Virus	Compounds Screened	Bacteria	Compounds Screened
	H3N2	2210,582	*Acinetobacter baumannii*	308,163
	H1N1	171,150	*Burkholderia cepacia*	579
	RSV	488,180	*Vibrio cholerae*	413,421
	Blue Tongue	214,017	*Escherichia coli*	319,519
	DenV	288,380	*Enterococcus faecalis*	580
	VEEV	406,323	*Enterococcus faecium*	580
	HIV	53,760	*Methicillin-resistant Staphylococcus aureus (MRSA)*	317,558
	HSV	187,452	*Staphylococcus aureus*	725,599
BSL 2	CMV	5120	*Salmonella enterica serovar Typhimurium (S. Typhimurium)*	269,088
	Zika	323,943	*Shigella dysenteriae*	579
			*Streptococcus pneumonia*	46,873
			*Pseudomonas Aeruginosa*	579
			*Mycobacterium tuberculosis H37Ra*	13,674
			*Yersinia pestis*	11,395
	**Total**	4348,907		2428,187
	H5N1	585,953	*Mycobacterium tuberculosis* H37Rv	1505,170
	WNV	562,211		
	SARS	369,732		
BSL3	Chik V	277,190		
	SARS-2	2400,487		
	MERS	2028		
	**Total**	4197,601		1505,170
BSL4*	Ebola	7026		
	Nipah	10,262		
	**Total**	17,288		

* BSL-4 screens were performed at University of Texas Medical Branch with experimental design input and data management by Southern Research.

**Table 3:** The average Z’ value for each assay that is discussed and the resulting average potency values for each reference compound. Note that the number of replicates for each reference is not equal due to their not being included in every performance of the assay. The higher variability of data associated with the inclusion of efflux inhibitor is discussed in the text.

Assay	Average Assay Z’	Chloroquine			Remdesivir			Calpain Inhibitor IV			PF-07321332+EI			PF-07321332		
		LogIC_50_ (M) (std dev)	IC_50_, μM	N	LogIC_50_ (M) (std dev)	IC_50_, μM	N	LogIC_50_ (M) (std dev)	IC_50_, μM	N	LogIC_50_ (M) (std dev)	IC_50_, μM	N	LogIC_50_ (M) (std dev)	IC_50_, μM	N
SARS-CoV-2 CPE (WA1/2020)	0.79	−5.38 (0.25)	4.17	101	−4.99 (0.21)	10.2	99	−6.61 (0.33)	0.25	102	~−7.36	~−0.044	32*	−5.56 (0.16)	2.75	40
SARS-CoV-2 Nanoluc (based on WA1/2020)	0.85	−5.55 (0.27)	2.82	82	−6.69 (0.30)	0.204	80	−7.87 (0.69)	0.013	80	ND	ND	ND	−7.07 (0.14)	0.085	71
SARS-CoV-2 CPE (Delta)	0.73	−5.40 (0.21)	3.98	10	−5.04 (0.22)	9.12	10	−6.84 (0.34)	0.145	10	−7.59 (0.20)	0.026	9	−5.57 (0.20)	2.69	9
SARS-CoV-2 CPE (Omicron)	0.72	−5.14 (0.20)	7.24	8	−5.04 (0.17)	9.12	8	−6.71 (0.28)	0.195	8	−7.38 (0.17)	0.042	7^†^	−5.62 (0.15)	2.40	8
SARS-CoV CPE (Toronto-2 strain)	0.72	−5.24 (0.20)	5.75	24	−4.95 (0.18)	11.2	23	−6.23 (0.45)	0.59	24	−7.42 (0.55)	0.038	8	−5.21 (0.17)	6.16	8
MERS CPE (Lineage C- Novel/2012)	0.65	~−4.57	~27	13^‡^	ND	ND	ND	−5.69 (0.23)	2.04	12	−7.26 (0.13)	0.055	8	ND	ND	ND

* 8 values not included due to cytotoxic effect of efflux inhibitor;

† one value removed due to cytotoxic effect of efflux inhibitor;

‡ low potency replicates assigned an IC_50_ of 30 μM for the approximate average calculation;

ND = Not Determined; EI = Efflux Inhibitor, CP-100356.

**Table 4:** Virus and cell lines used in the cell-based coronavirus assays.

Reagent	Description	Source	Length of assay (type of assay)
SARS-CoV-2 virus	USA_WA1/2020	World Reference Center for Emerging Viruses and Arboviruses (WRCEVA)	72 h (CPE)
SARS-CoV-2 virus	Delta	BEI Resources; Catalog No. NR-55,674	72 h (CPE)
SARS-CoV-2 virus	Omicron	BEI Resources; Catalog No. NR-56,462	72 h (CPE)
SARS-CoV-2 reporter virus	Based on USA_WA1/2020	Licensed from the University of North Carolina, Chapel Hill.	72 h (reporter virus)
SARS-CoV	Toronto-2	Gifted by Dr. Heinz Feldman	72 h (CPE)
MERS-CoV	Human Beta-Coronavirus Lineage C –Novel/2012	Erasmus Medical Center	96 h (CPE)
Vero CCL-81	kidney tissue derived from a normal, adult African green monkey	ATCC.org	N/A
Vero E6	Vero cells sub-cloned for high expression of ACE-2 receptor	Obtained from laboratory of Ralph Baric, University of North Carolina, Chapel Hill	N/A
A549-ACE-2	A549 cells expressing ACE-2 receptor	Obtained from laboratory of Ralph Baric, University of North Carolina, Chapel Hill	N/A

## Abbreviations:

BSL-3: biosafety level 3
SR: Southern Research Institute
MERS-CoV: Middle East Respiratory Syndrome
HTS: High-Throughput Screening
SAR: structure-activity relationship
RSV: Respiratory syncytial virus
BIV: bovine immunodeficiency virus
HIV: human immunodeficiency virus
SARS-CoV: Severe Acute Respiratory Syndrome Coronavirus
CPE: cytopathic effect
ARPs: Assay Ready Plates
ACE-2: angiotensin-converting enzyme 2

## References

[1] Johnson NP, Mueller J. Updating the accounts: global mortality of the 1918–1920 “Spanish” influenza pandemic. Bull Hist Med 2002;76(1):105–15. 11875246 10.1353/bhm.2002.0022

[2] Msemburi W, The WHO estimates of excess mortality associated with the COVID-19 pandemic. Nature 2023;613(7942):130–7. 36517599 10.1038/s41586-022-05522-2 PMC9812776

[3] Pustake M, SARS, MERS and CoVID-19: an overview and comparison of clinical, laboratory and radiological features. J Family Med Prim Care 2022;11(1): 10–7. 35309670 10.4103/jfmpc.jfmpc_839_21 PMC8930171

[4] Gupta S, Rouse BT, Sarangi PP. Did climate change influence the emergence, transmission, and expression of the COVID-19 pandemic? Front Med (Lausanne) 2021;8:769208. 34957147 10.3389/fmed.2021.769208 PMC8694059

[5] Rasmussen L, Adapting high-throughput screening methods and assays for biocontainment laboratories. Assay Drug Dev Technol 2015;13(1):44–54. 25710545 10.1089/adt.2014.617 PMC4340648

[6] Severson WE, Development and validation of a high-throughput screen for inhibitors of SARS CoV and its application in screening of a 100,000-compound library. J Biomol Screen 2007;12(1):33–40. 17200104 10.1177/1087057106296688 PMC9050465

[7] Ananthan S, High-throughput screening for inhibitors of Mycobacterium tuberculosis H37Rv. Tuberculosis (Edinb) 2009;89(5):334–53. 19758845 10.1016/j.tube.2009.05.008 PMC3255569

[8] Huryn DM, Cosford NDP. Chapter 26 the molecular libraries screening center network (MLSCN): identifying chemical probes of biological systems. Annu Rep Med Chem 2007;42:401–16. 32287469 10.1016/S0065-7743(07)42026-7 PMC7112292

[9] Kannon M, A novel approach to identify inhibitors of iron acquisition systems of pseudomonas aeruginosa. Microbiol Spectr 2022;10(5):e0243722. 36098531 10.1128/spectrum.02437-22 PMC9604216

[10] Emert-Sedlak LA, Inhibitors of HIV-1 Nef-mediated activation of the myeloid src-family kinase hck block HIV-1 replication in macrophages and disrupt MHC-I downregulation. ACS Infect Dis 2022;8(1):91–105. 34985256 10.1021/acsinfecdis.1c00288 PMC9274903

[11] Haese NN, Identification of quinolinones as antivirals against venezuelan equine encephalitis virus. Antimicrob Agents Chemother 2021;65(9):e0024421. 34152810 10.1128/AAC.00244-21 PMC8373297

[12] Ahmed SK, Targeting chikungunya virus replication by benzoannulene inhibitors. J Med Chem 2021;64(8):4762–86. 33835811 10.1021/acs.jmedchem.0c02183 PMC9774970

[13] Dalecki AG, High-throughput screening and Bayesian machine learning for copper-dependent inhibitors of Staphylococcus aureus. Metallomics 2019;11(3): 696–706. 30839007 10.1039/c8mt00342d PMC6467072

[14] Pery E, Identification of a novel HIV-1 inhibitor targeting Vif-dependent degradation of human APOBEC3G protein. J Biol Chem 2015;290(16):10504–17. 25724652 10.1074/jbc.M114.626903 PMC4400358

[15] Tigabu B, A BSL-4 high-throughput screen identifies sulfonamide inhibitors of Nipah virus. Assay Drug Dev Technol 2014;12(3):155–61. 24735442 10.1089/adt.2013.567 PMC3994909

[16] Chung DH, Discovery of a novel compound with anti-venezuelan equine encephalitis virus activity that targets the nonstructural protein 2. PLoS Pathog 2014;10(6):e1004213. 24967809 10.1371/journal.ppat.1004213 PMC4072787

[17] Reynolds RC, High throughput screening of a library based on kinase inhibitor scaffolds against Mycobacterium tuberculosis H37Rv. Tuberculosis (Edinb) 2012;92(1):72–83. 21708485 10.1016/j.tube.2011.05.005 PMC3183257

[18] White EL, Discovery and Development of Highly Potent Inhibitors of Mycobacterium tuberculosis Growth In Vitro. In: Probe Reports from the NIH Molecular Libraries Program; 2010. 23762923

[19] Chung DH, HTS-driven discovery of new chemotypes with West Nile Virus inhibitory activity. Molecules 2010;15(3):1690–704. 20336008 10.3390/molecules15031690 PMC4839297

[20] Maddry JA, Antituberculosis activity of the molecular libraries screening center network library. Tuberculosis (Edinb) 2009;89(5):354–63. 19783214 10.1016/j.tube.2009.07.006 PMC2792876

[21] Li Q, Assay development and high-throughput antiviral drug screening against Bluetongue virus. Antiviral Res 2009;83(3):267–73. 19559054 10.1016/j.antiviral.2009.06.004 PMC2727572

[22] Severson WE, High-throughput screening of a 100,000-compound library for inhibitors of influenza A virus (H3N2). J Biomol Screen 2008;13(9):879–87. 18812571 10.1177/1087057108323123 PMC2782602

[23] Noah JW, A cell-based luminescence assay is effective for high-throughput screening of potential influenza antivirals. Antiviral Res 2007;73(1):50–9. 16904762 10.1016/j.antiviral.2006.07.006

[24] Martis EA, Badve RR. High-Throughput Screening: the Hits and Leads of Drug Discovery - An Overview. J Appl Pharm Sci 2011;1(1):2–10.

[25] Szymański P, Markowicz M, Mikiciuk-Olasik E. Adaptation of high-throughput screening in drug discovery-toxicological screening tests. Int J Mol Sci 2012;13(1): 427–52. 22312262 10.3390/ijms13010427 PMC3269696

[26] Martinez-Gzegozewska Y, High-Throughput cell-based immunofluorescence assays against influenza. SLAS Discov 2024;29(1):66–76. 37925159 10.1016/j.slasd.2023.10.008 PMC10859970

[27] Sastre P, Oomens AG, Wertz GW. The stability of human respiratory syncytial virus is enhanced by incorporation of the baculovirus GP64 protein. Vaccine 2007;25 (27):5025–33. 17544182 10.1016/j.vaccine.2007.04.066 PMC2593139

[28] Collins PL, Fearns R, Graham BS. Respiratory syncytial virus: virology, reverse genetics, and pathogenesis of disease. Curr Top Microbiol Immunol 2013;372: 3–38. 24362682 10.1007/978-3-642-38919-1_1 PMC4794264

[29] Ausar SF, High-throughput screening of stabilizers for respiratory syncytial virus: identification of stabilizers and their effects on the conformational thermostability of viral particles. Hum Vaccin 2007;3(3):94–103. 17426457 10.4161/hv.3.3.4149

[30] Gonda MA, Characterization and molecular cloning of a bovine lentivirus related to human immunodeficiency virus. Nature 1987;330(6146):388–91. 3683555 10.1038/330388a0

[31] Rasmussen L, A high-throughput screening strategy to overcome virus instability. Assay Drug Dev Technol 2011;9(2):184–90. 21050067 10.1089/adt.2010.0298 PMC3065722

[32] Noah JW, Identification of a Series of Quinazolinediones as Potent, Selective, Post-Entry Inhibitors of Human Respiratory Syncytial Virus (hRSV) via a Cell-Based High Throughput Screen and Chemical Optimization. In: Probe Reports from the NIH Molecular Libraries Program; 2010. 23658939

[33] Moore BP, (S)-N-(2,5-Dimethylphenyl)-1-(quinoline-8-ylsulfonyl)pyrrolidine-2-carboxamide as a small molecule inhibitor probe for the study of respiratory syncytial virus infection. J Med Chem 2012;55(20):8582–7. 23043370 10.1021/jm300612z PMC3506029

[34] Mathieu E., Coronavirus Pandemic (COVID-19). 2020: https://ourworldindata.org/coronavirus.

[35] Cella E, Early Emergence Phase of SARS-CoV-2 Delta Variant in Florida, US. Viruses 2022;14(4). 10.3390/v14040766 PMC9028683 35458495

[36] Chu H, Comparative tropism, replication kinetics, and cell damage profiling of SARS-CoV-2 and SARS-CoV with implications for clinical manifestations, transmissibility, and laboratory studies of COVID-19: an observational study. Lancet Microbe 2020;1(1):e14–23. 32835326 10.1016/S2666-5247(20)30004-5 PMC7173822

[37] Hou YJ, SARS-CoV-2 reverse genetics reveals a variable infection gradient in the respiratory tract. Cell 2020;182(2):429–46. e14. 32526206 10.1016/j.cell.2020.05.042 PMC7250779

[38] Owen DR, An oral SARS-CoV-2 M(pro) inhibitor clinical candidate for the treatment of COVID-19. Science 2021;374(6575):1586–93. 34726479 10.1126/science.abl4784

[39] Syed YY. Molnupiravir: first Approval. Drugs 2022;82(4):455–60. 35184266 10.1007/s40265-022-01684-5 PMC8858220

[40] Barnard DL, Inhibition of severe acute respiratory syndrome-associated coronavirus (SARSCoV) by calpain inhibitors and beta-d-N4-hydroxycytidine. Antivir Chem Chemother 2004;15(1):15–22. 15074711 10.1177/095632020401500102

[41] Colson P, Chloroquine and hydroxychloroquine as available weapons to fight COVID-19. Int J Antimicrob Agents 2020;55(4):105932. 32145363 10.1016/j.ijantimicag.2020.105932 PMC7135139

[42] Hu H, Optimization of the prodrug moiety of remdesivir to improve lung exposure/selectivity and enhance anti-SARS-CoV-2 activity. J Med Chem 2022;65 (18):12044–54. 36070561 10.1021/acs.jmedchem.2c00758

[43] Xie X, A nanoluciferase SARS-CoV-2 for rapid neutralization testing and screening of anti-infective drugs for COVID-19. Nat Commun 2020;11(1):5214. 33060595 10.1038/s41467-020-19055-7 PMC7567097

[44] Gorshkov K, The SARS-CoV-2 cytopathic effect is blocked by lysosome alkalizing small molecules. ACS Infect Dis 2021;7(6):1389–408. 33346633 10.1021/acsinfecdis.0c00349 PMC7771250

[45] Chen CZ, Drug repurposing screen for compounds inhibiting the cytopathic effect of SARS-CoV-2. Front Pharmacol 2020;11:592737. 33708112 10.3389/fphar.2020.592737 PMC7942396

[46] Murgolo N, SARS-CoV-2 tropism, entry, replication, and propagation: considerations for drug discovery and development. PLoS Pathog 2021;17(2): e1009225. 33596266 10.1371/journal.ppat.1009225 PMC7888651

[47] Kim S, Differential interactions between human ACE2 and Spike RBD of SARS-CoV-2 variants of concern. J Chem Theory Comput 2021;17(12):7972–9. 34856802 10.1021/acs.jctc.1c00965 PMC8672429

[48] Kalgutkar AS, N-(3,4-dimethoxyphenethyl)-4-(6,7-dimethoxy-3,4-dihydroisoquinolin-2[1H]-yl)-6,7-dimethoxyquinazolin-2-amine (CP-100356) as a “chemical knock-out equivalent” to assess the impact of efflux transporters on oral drug absorption in the rat. J Pharm Sci 2009;98(12):4914–27. 19373887 10.1002/jps.21756

[49] Zhu Y, Generation of a VeroE6 Pgp gene knock out cell line and its use in SARS-CoV-2 antiviral study. Antiviral Res 2022;208:105429. 36208677 10.1016/j.antiviral.2022.105429 PMC9533647

[50] Wachira LJ, Why are COVID-19 effects less severe in sub-saharan Africa? Moving more and sitting less may be a primary reason. Prog Cardiovasc Dis 2022; 71:103–5. 35487264 10.1016/j.pcad.2022.04.012 PMC9042414

[51] Levin AT, Assessing the burden of COVID-19 in developing countries: systematic review, meta-analysis and public policy implications. BMJ Glob Health 2022;7(5). 10.1136/bmjgh-2022-008477 PMC9136695 35618305

[52] Woodruff J, Warsi Z. COVID rapidly spreads in China as government eases strict quarantine rules. In: PBS News Hour. Public Broadcasting Service; 2022. https://www.pbs.org/newshour/show/covid-rapidly-spreads-in-china-as-government-eases-strict-quarantine-rules.

[53] NIAID. NIAID announces antiviral drug development awards. National Institute of Allergy and Infectious Diseases; 2022.

[54] Public health and social measures for health emergencies and pandemics in the EU/EEA: recommendations for strengthening preparedness planning 2024 Report, European Centre for Disease Prevention and Control, Stockholm.

